# Effect of miR-206 on lower limb ischemia–reperfusion injury in rat and its mechanism

**DOI:** 10.1038/s41598-023-48858-z

**Published:** 2023-12-07

**Authors:** Hui Wang, Meng-Jie Shi, Zhang-Qin Hu, Lin Miao, He-Shi Cai, Rui-Peng Zhang

**Affiliations:** 1 Department of Vascular Surgery, Shaanxi Provincial People’s Hospital, 256 Youyixi Road, Xi’an, 710068 Shaanxi China; 2 Department of Cardiovascular Surgery, Shaanxi Provincial People’s Hospital, 256 Youyixi Road, Xi’an, 710068 Shaanxi China

**Keywords:** Cell biology, Molecular biology, Biomarkers

## Abstract

Lower limb ischemia–reperfusion is a common pathological process during clinical surgery. Because lower limb ischemia–reperfusion usually aggravates ischemia-induced skeletal muscle tissue injury after lower limb ischemia–reperfusion, it also causes remote organ heart, intestine, liver, lung and other injuries, and there is no effective clinical treatment for lower limb ischemia–reperfusion injury, so it is urgent to study its injury mechanism. In this study, the rat model of lower limb ischemia–reperfusion was established by clamping the femoral artery with microarterial clips, and the wall destruction such as intimal injury, cell edema, collagen degeneration, neutrophil infiltration, and elastic fiberboard injury of the femoral artery wall was detected. The expression of inflammatory factors was detected by immunohistochemistry. miR-206 preconditioning was used to observe the expression of inflammatory factors, redox status and apoptosis in the vascular wall of rats after acute limb ischemia–reperfusion. Our findings suggest that vascular endothelial cell edema increases, wall thickening, neutrophil infiltration, and elastic fiber layer damage during IRI. Inflammatory factor expression was increased in femoral artery tissue, and miR-206 expression levels were significantly down-regulated. Further studies have found that miR-206 attenuates lower limb IRI by regulating the effects of phase inflammatory factors. In this study, we investigated the effect of miR-206 on inflammatory factors and its possible role in the development of lower limb IRI, providing new research ideas for the regulatory mechanism of lower limb IRI, and providing a certain theoretical basis for the treatment of lower limb ischemia–reperfusion injury after surgery or endovascular intervention.

## Introduction

Ischemia–reperfusion injury (IRI) is a pathological damage of its structure and function caused by excessive ischemic time of tissues and organs, and more serious damage caused by ischemic tissues and organs after restoring blood supply^1^. Acute limb ischemia (ALI) is one of the most common critical ischemic diseases in vascular surgery, with dramatic onset and poor prognosis, often caused by sudden interruption of arterial vascular blood flow^2^. The most common clinical causes are cardiogenic embolus detachment, arterial thrombosis (AE), arterial vascular compression and arterial continuity interruption, of which atrial fibrillation cardiogenic embolus detachment is the most common^3^.

In recent years, with the rapid development of interventional techniques in vascular surgery and the application of mechanical thrombectomy devices, acute lower limb arterial ischemic diseases in clinical practice can be treated in time, and the clinical prognosis is significantly improved, but ALI still has a high amputation disability rate, and ischemia–reperfusion injury has become a severe problem to be solved in vascular shell letters^4^. The mechanisms of ischemia–reperfusion injury are: acute inflammatory reaction of ischemic wall, vascular endothelial cell injury, wall edema, thrombosis, lumen occlusion, and organ injury. Eventually lead to rhabdomyolysis, lower limb compartment syndrome, etc., a large number of toxins released into the blood, severe cases can lead to metabolic acidosis, hyperkalemia, multiple organ failure, and even death^5,6^. According to the traditional concept, skeletal muscle cells belong to terminally differentiated cells and have no ability to redivide, so skeletal muscle injury is considered irreversible. In muscle satellite cell regeneration, many miRNA families play regulatory roles in this complex network, such as the miR-206 family^7–9^.

MiRNAs are non-coding RNA molecules of approximately 22 nucleotides in length, which can negatively regulate target genes at the post-transfer level by means of mRNA cleavage or inhibition of protein translation^10^. The miR-206 family belongs to myogenic miRNAs and was first cloned and identified from human and mouse muscle tissues, and its coding gene is localized on chromosome 6, and gene chip detection revealed that miR-206 presents spatiotemporal specific expression during skeletal muscle cell differentiation and maturation, which suggests that miR-206 may have a role in contributing to muscle differentiation^11,12^.

Although miR-206 has been increasingly studied during skeletal muscle development, few studies have been reported in limb ischemia–reperfusion injury. In this study, we constructed a rat model of lower limb ischemia–reperfusion injury and evaluated the effect of the model. Through miR-206 preconditioning, to investigate the potential molecular mechanism of miR-206 in regulating lower limb ischemia–reperfusion injury in rats, and to provide an important theoretical basis for limb ischemia–reperfusion injury and explore possible therapeutic targets.

## Materials and methods

### Animal study

This study was approved by the Ethics Committee of Shaanxi Provincial People’s Hospital, China. We confirmed that all experiments were performed in accordance with relevant guidelines and regulations. We also complied with the requirements of ARRIVE guidelines concerning live animal research. Twenty-four SPF grade male SD rats aged 42 -56 days were purchased from Hubei Laboratory Animal Research Center. Twenty-four male SD rats were randomly divided into four groups: (i) Sham group (n = 6), (ii) IRI group (n = 12, IRI 6 h = 3, IRI 12 h = 3, IRI 24 h = 6), (iii) IRI + miR-206 agomir group (n = 3) and (iv) IRI + miR-206 antagomir group (n = 3). Isoflurane was used for anesthesia, 5% induction anesthesia, 2% maintenance anesthesia, and 1 L/min gas flow. After the rats were anesthetized in the supine position, the hair on the right lower side of the rats was shaved, moistened with alcohol, and a longitudinal incision was made along the inner thigh, approximately 1 cm in length. Femoral arteries were dissected and clamped with hemoclips for reperfusion after 4 h of ischemia. In the sham group, only the femoral artery was freed without clamping the femoral artery. Femoral arteries were sampled at 6 h, 12 h, and 24 h of reperfusion after 4 h of ischemia, and each femoral artery was bisected, half fixed, and half cryopreserved. MiR-206 preconditioning group + ischemia–reperfusion group (miR-206 + IRI group): femoral artery was freed and clamped, and reperfusion treatment was performed after 4 h of ischemia, while miR-206-related reagents were injected into the tail vein. Blood samples were collected from the lower limb veins of all rats after 24 h of reperfusion; the rats were sacrificed by carotid artery exsanguination 24 h after reperfusion, and the right hind limb muscle tissue and femoral artery were harvested, some of which were stored at − 80 °C for future use, and some of which were stored in 4% paraformaldehyde for fixation and tissue section staining was done. Laser Doppler was used for measuring blood flow.

### HE staining

The slices were washed in xylene I 10 min—xylene II 10 min—anhydrous ethanol I 5 min—anhydrous ethanol II5 min—95% alcohol 5 min—90% alcohol 5 min—80% alcohol 5 min—70% alcohol 5 min—distilled water successively for dewaxing treatment. Then the nucleus was stained with hematoxylin, the slices were injected into the cells and stained with hematoxylin for 3–8 min, washed with tap water, differentiated with 1% hydrochloric acid alcohol for several seconds, rinsed with tap water, returned blue with 0.6% ammonia water, and rinsed with running water. The sections were stained in eosin dye solution for 1–3 min for cytoplasmic staining. The sections were then dehydrated and transparent in 95% alcohol I 5 min—95% alcohol II 5 min—anhydrous ethanol I 5 min—anhydrous ethanol II 5 min—xylene I5 min—xylene II 5 min, and the sections were taken out of xylene to dry slightly and sealed with neutral gum. Microscopic examination, image acquisition and analysis. RESULTS: the nucleus was blue, and the cytoplasm, muscle fibers, collagen fibers and red blood cells were red in different degrees, which were mainly used to observe the histomorphological structure.

### Immunohistochemistry

Paraffin sections were sequentially placed in xylene I (20 min)—xylene II (20 min)—xylene III (20 min)—absolute ethanol I (5 min)—absolute ethanol II (5 min)—95% alcohol (5 min)—90% alcohol (5 min)—80% alcohol (5 min)—70% alcohol (5 min), followed by distilled water immersion for 5 min. Antigen retrieval was performed using an electric pottery furnace. The deparaffinized and hydrated tissue sections were placed on a heat-resistant plastic section rack in a beaker (or retrieval box), and an appropriate amount of retrieval solution (0.01 M citrate buffer, pH 6.0) was added into the beaker for 15 min. After that, the beaker was removed from the microwave furnace and placed in cold water to cool down. When the retrieval solution dropped to room temperature, the slides were removed and rinsed three times with PBS (pH 7.4) for 3 min each time.Prepared 3% hydrogen peroxide was dropped onto the sectioned tissue to block endogenoÙus peroxidase, incubated at room temperature for 15 min, and rinsed three times with PBS for 3 min each time.The slides were dried with absorbent paper, and the brush of the immunized group was circled around the tissue, and diluted normal rabbit serum was added dropwise and blocked at room temperature for 30 min to reduce non-specific staining.Excess fluid was shaken off and not washed, then diluted primary antibodies (HMGB1 1:100, TNF-α 1:100, IL-6 1:100) were added dropwise, and incubated overnight at 4 °C in a humidified chamber after adding the primary antibody.Sections were rinsed three times with PBS for 3 min, dried with absorbent paper and then instilled with biotinylated secondary antibody, incubated at 37 °C for 30 min in a humidified chamber, and rinsed three times with PBS for 3 min each.After the sections were dried with absorbent paper, SABC complex was dropped and incubated at 37 °C for 30 min in a humidified chamber, and the sections were rinsed four times with PBS for 3 min each time. The sections were rinsed with PBS 4 times for 3 min each time, the PBS solution was shaken off, the sections were dried with absorbent paper, and freshly prepared DAB chromogenic solution was added dropwise to each section and observed microscopically, and the positive signal was brownish-yellow or tan, and the color development was terminated by rinsing the sections with tap water when it was felt possible. Mayer’s hematoxylin counterstaining was performed at regular intervals for 2 min before reversion to blue with PBS water. After rinsing the sections in water, they were dehydrated and cleared in 70% alcohol-80% alcohol-90% alcohol-95% alcohol-absolute ethanol I-absolute ethanol II-absolute ethanol II-xylene I-xylene II in turn, placed in each reagent for 5 min, and finally air-dried in a hood. Drop neutral gum next to the tissue and cover with a coverslip, lay flat on one side first, then gently lower the other side to avoid air bubbles, and place the sealed section flat in a hood to dry. Air-dried sections can be observed or images acquired under a microscope. Interpretation of results: Blue is the nucleus, brownish-yellow or tan is the target protein expression.

### Quantitative real-time PCR

RNA was extracted by Trizol method (Ambion, Texas, USA), and OD260, OD280, and OD260/OD280 values were measured with a microspectrophotometer to calculate the purity and concentration of RNA. RNA quality was estimated based on OD260/OD280 ratios, which met experimental requirements between 1.8 and 2.0.RT was reverse transcribed into cDNA, and the reaction system was detected by real-time PCR as follows: 0.4 μl Forward Primer (10 μM), 0.4 μl Reverse Primer (10 μM), 10 μl SYBR Green Master Mix, 0.4 μl 50 × ROX Reference Dye 2, and 4.8 μl H_2_O.Primer sequences were: U6 Forward: CCCTGGAGAAGAGCTACGAG, U6 Reverse: ACACGTGGTCTTTGCGGATG; miR-206 Forward: TGCGCTGGAATGTAAGGAAGTG, miR-206 Reverse: CCAGTGCAGGGTCCGAGGTATT. **Western blot analyses** The extracted protein supernatant was mixed with 5 times protein loading buffer (volume ratio 4:1) and placed in boiling water for boiling water bath for 10 min. After denaturation, it was cooled to room temperature and then stored at − 20 °C. The prepared protein samples and MAKER were added to the loading wells with a micropipette, and the total protein amount of each sample was 40 μg. Electrophoresis, transfer membrane. PVDF membranes were blocked by soaking in TBST (blocking solution) containing 5% nonfat dry milk, and the corresponding primary antibodies (GAPDH 1:1000, TNF-a 1:500, HMGB1 1:1000, IL-6 1:1000) were diluted in blocking solution to soak the PVDF membranes in primary antibody incubation solution and incubated overnight at 4 °C. TBST thoroughly washed the PVDF membrane 5 times, 5 min/time. The corresponding HRP-labeled secondary antibody (1:600) was diluted with TBST to soak the PVDF membrane in the secondary antibody incubation solution and incubated in a shaker at room temperature for 2 h. TBST thoroughly washed the PVDF membrane 5 times, 5 min/time. Mix the enhancement solution in ECL reagent with stable peroxidase solution in the proportion of 1:1, drop the working solution on PVDF membrane, react for several minutes, after the fluorescence band is obvious, suck off the excess substrate solution with filter paper, cover the plastic wrap, put the developing solution for development after X-ray film compression, fix the fixing solution for development, and rinse the film.Films were dried, scanned, and analyzed for film gray values with BandScan.

### TUNEL

Paraffin sections were sequentially placed in xylene I (20 min)—xylene II (20 min)—xylene III (20 min)—absolute ethanol I (5 min)—absolute ethanol II (5 min)—95% alcohol (5 min)—90% alcohol (5 min)—80% alcohol (5 min)—70% alcohol (5 min), followed by distilled water immersion for 5 min. The excess liquid on the slide was carefully removed with filter paper, and then proteinase K working solution (20 μg/ml) was dropped and applied at 37 °C for 20 min and washed twice with PBS for 5 min each time. Sections were then fixed by immersion in 4% paraformaldehyde (pH 7.4) solution for 5 min at room temperature and subsequently washed twice with PBS for 5 min each. For the above processed tissue sections, the excess liquid on the slides was carefully removed with absorbent paper, and 100 μL of Equilibration Buffer was dropped on the sample area and equilibrated at room temperature for 10 min. At the time of slide equilibration, the biotinylated nucleoside mixture was thawed on ice while preparing enough TdT enzyme reaction solution (98 µl Equilibratin Buffer + 1 µl Biotinylated Nucleotide + 1 µl TdT Enzyme) and stored on ice until use, and 100 ul of reaction solution per section was sufficient to cover the sample area.Carefully suck off the excess liquid around the sample area with absorbent paper, drop 100 μL of TdT enzyme reaction solution on the sample area, do not let the sample dry, cover the plastic cover slip on the reaction area to ensure that the reaction solution is evenly distributed, incubate in a humid box at 37 °C for 60 min, so that the end labeling reaction occurs.Dilute 20X SSC 1:10 in DI water.The coverslips were removed, and the reaction was terminated by placing the slides in a staining jar with 2X SSC solution, left at room temperature for 15 min, and then immersed in PBS three times for 5 min at room temperature.Endogenous catalase was inhibited by immersion in 0.3% hydrogen peroxide for 5 min and washed three times with PBS for 5 min each.100 µl Streptavidin HRP (streptavidin horseradish peroxidase) solution diluted 1:500 in PBS was added to the samples and incubated at room temperature for 30 min and washed three times with PBS for 5 min each time.100 µl DAB chromogen solution was added to the sample and color development was performed at room temperature for 2 min. After observing the color change under a microscope, the color development was terminated by rinsing with distilled water. Mayer ‘s hematoxylin counterstaining was performed at regular intervals for 2 min before reversion to blue with PBS water.After rinsing the sections in water, the sections were dehydrated and cleared in 70% alcohol-80% alcohol-90% alcohol-95% alcohol-absolute ethanol I-absolute ethanol II-absolute ethanol II-xylene I-xylene II successively, placed in each reagent for 5 min, and finally air-dried in a fume hood. Drop the neutral gum next to the tissue and cover with a coverslip, lay flat on one side first, then gently put down the other side to avoid bubbles, and lay flat in the hood to dry the sealed sections. Air-dried sections can be observed or images acquired under a microscope.Result determination: Apoptotic cells were brown and nuclei were blue or blue-black on histological sections under microscope.

### SOD assay

The tissues were rinsed with pre-chilled PBS (0.01 M, pH = 7.4) to remove residual blood, weighed and then minced. Cut the minced tissue and corresponding volume of PBS (generally according to the weight/volume ratio of 1:9, for example, 1 g of tissue sample corresponds to 9 mL of PBS, the specific volume can be appropriately adjusted according to the experimental needs, and recorded. Finally, the homogenate was centrifuged at 5000 × g for 10 min at 4 °C, and the supernatant was taken for detection. Total superoxide dismutase (SOD) test kit (Nanjing Jiancheng Bioengineering Institute) was used for detection according to the instructions.

### MDA assay

The tissues were rinsed with pre-chilled PBS (0.01 M, pH = 7.4) to remove residual blood, weighed and then minced. Cut the minced tissue and corresponding volume of PBS (generally according to the weight/volume ratio of 1:9, for example, 1 g of tissue sample corresponds to 9 mL of PBS, the specific volume can be appropriately adjusted according to the experimental needs, and recorded. Finally, the homogenate was centrifuged at 5000 × g for 10 min at 4 °C, and the supernatant was taken for detection. Malondialdehyde (MDA) test kit (Nanjing Jiancheng Bioengineering Institute) was used to detect according to the instructions.

### ROS staining

The tissues were rinsed with pre-chilled PBS (0.01 M, pH = 7.4) to remove residual blood, weighed and then minced. Cut the minced tissue and corresponding volume of PBS. The minced tissue samples were digested using collagenase IV/V digestion solution to prepare single-cell suspensions. Reactive Oxygen Species Assay Kit (Beyotime, Shanghai, China) was used to detect ROS production according to the instructions.

### Statistical analysis

SPSS 18.0 was used to analyse the data. Bonferroni test and two-way analysis was used to compare the haemodynamic parameters. One-way ANOVA was used to compare biochemical parameters between groups. A p < 0.05 was considered as statistically significant.

## Results

### Establishment of lower limb ischemia–reperfusion model in rats

**Figure 1:**
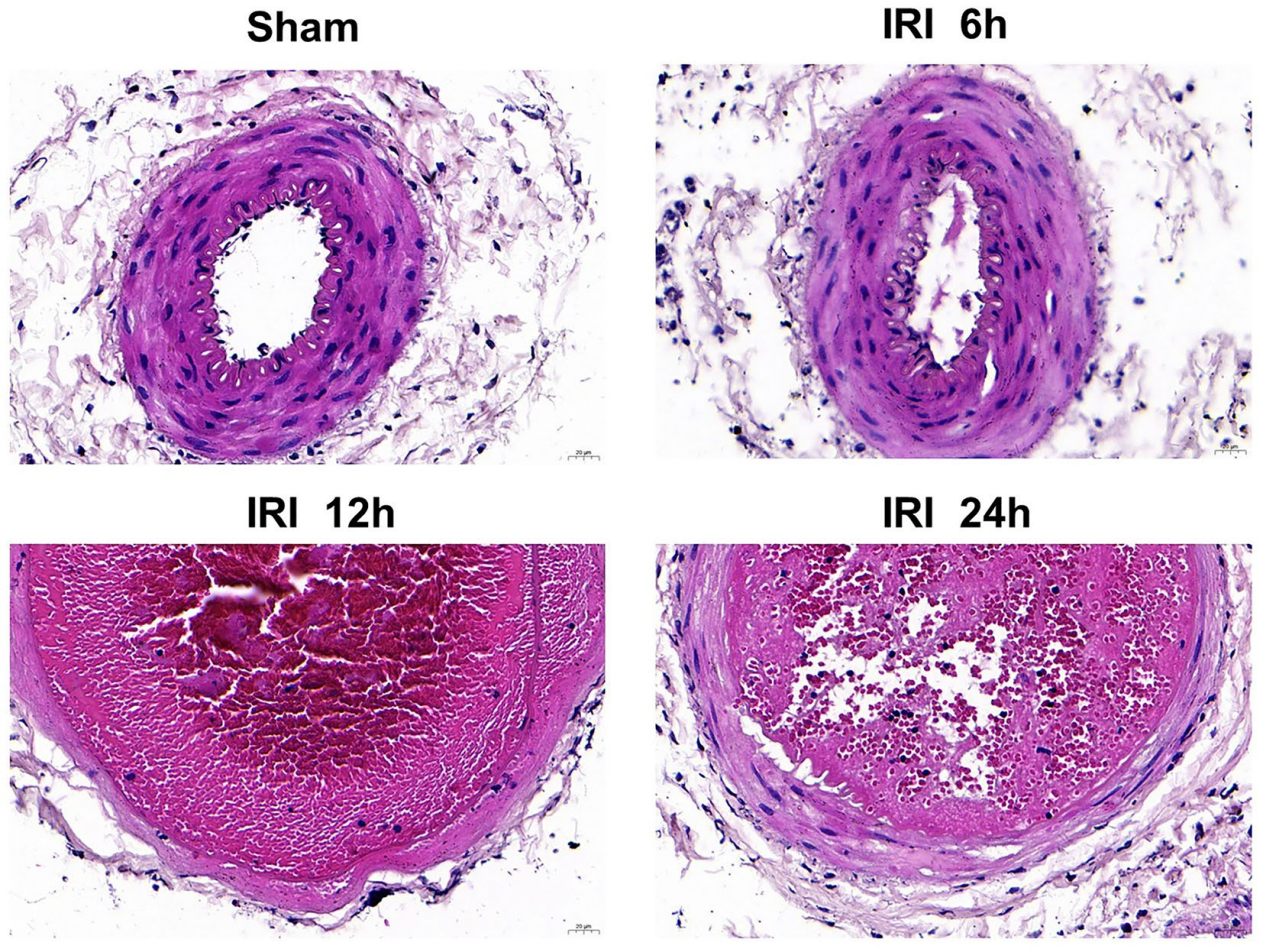
Establishment of lower limb ischemia–reperfusion model in rats**.** (**A**) Histomorphological analysis of femoral artery in each group (400 ×)..

Classical proximal femoral artery and distal femoral artery ligation sites were used to establish the model. When the distal femoral artery blood pressure was measured to be 0, it indicated that the ischemic model was successful. After 4 h of ischemia, the arterial clamp was removed to restore the lower limb blood flow. At this time, the measured blood pressure increase marker reperfusion was successful. After the completion, the wound was sutured. HE staining was observed under light microscope: the structure of the three-layer membrane of the femoral artery in the sham group was clear and intact. In IRI group, the right femoral artery wall was sectioned and observed under light microscope: the three membrane structures were thickened to varying degrees and the boundaries were blurred. Intima: Increased endothelial cell size, wall detachment, few platelets adhered. Subendothelial: Numerous inflammatory cell infiltrates. Medial: massive inflammatory cell infiltration, irregular smooth muscle cell morphology, fiberboard rupture, occasional cell foamy degeneration. Adventitia: edema and thickening of fibrous connective tissue, local rupture and defect. This response became increasingly severe with longer reperfusion times (Fig. 1).

### MiR-206 inhibits ischemia–reperfusion injury in rat lower limb

**Figure 2:**
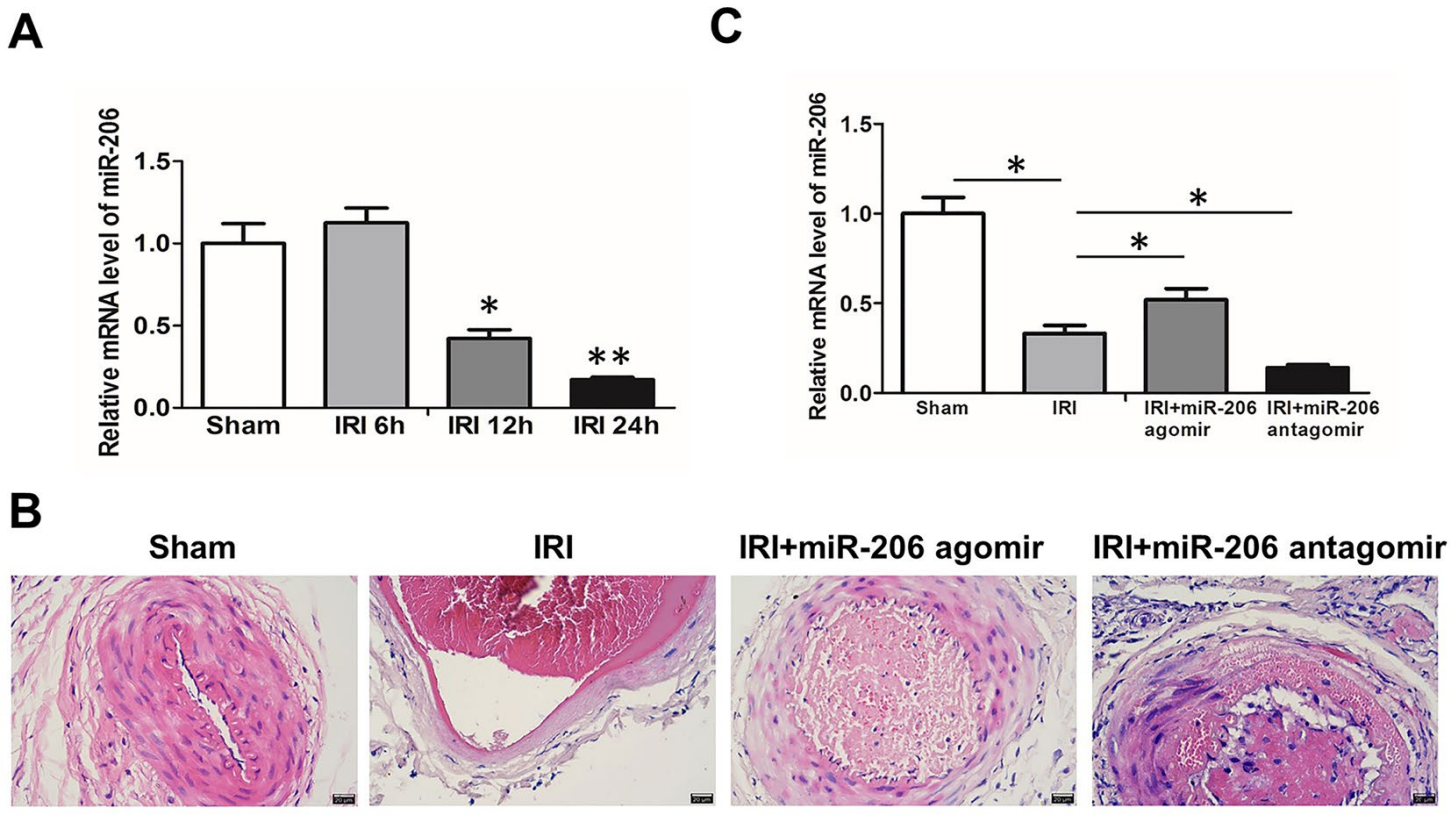
MiR-206 inhibits ischemia–reperfusion injury in rat lower limb. (**A**) qRT-PCR results of miR-206 in each group. (**B**) Histomorphological analysis of femoral artery in each group (400 ×).(C) qRT-PCR results of miR-206 in each group. All experiments were duplicated for three times. Values are mean ± standard deviation. *, *p* < 0.05. **, *p* < 0.01.

After establishing the rat lower limb ischemia–reperfusion model, qRT-PCR results showed that miR-206 expression levels were significantly down-regulated over time in the IRI group (Fig. 2A). miR-206 agomir and miR-206 anagomir were injected into the tail vein for treatment. The results of HE staining suggested that the structure of the three-layer membrane of the femoral artery was clear and intact and normal in the sham group. In the IRI group, endothelial cells increased in size, the wall was detached, and a small amount of platelets adhered. Large subendothelial inflammatory cell infiltrates. A large number of inflammatory cells infiltrated in the media, smooth muscle cells were irregular in shape, the fiberboard was broken, and foam degeneration of cells was occasionally observed. Fibrous connective tissue in the adventitia was edematous and thickened, and locally broken and defective. Compared with the IRI group, the degree of endothelial cell edema, wall thickening, neutrophil infiltration, and elastic fiber layer injury were alleviated in the miR-206 agomir + IRI group, while the above conditions were significantly aggravated in the miR-206 anagomir + IRI group (Fig. 2B). qRT-PCR results indicated that miR-206 expression levels were significantly down-regulated in the IRI group, increased after miR-206 agomir treatment, and significantly down-regulated after miR-206 anagomir treatment (Fig. 2C).

### MiR-206 inhibits ischemia–reperfusion injury in rat lower limb via oxidative stress

**Figure 3:**
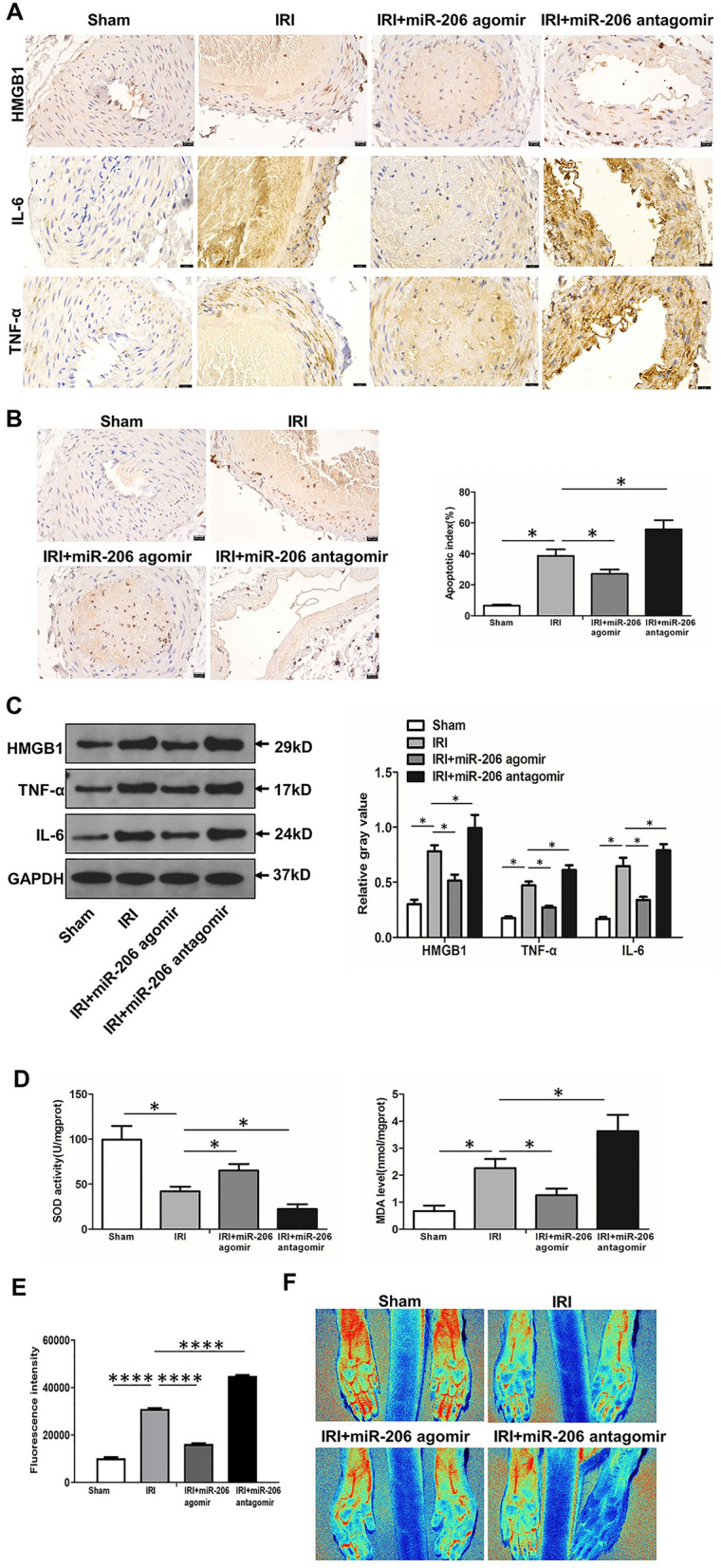
MiR-206 inhibits ischemia–reperfusion injury in rat lower limb. (**A**) Immunohistochemical staining of HMGB1, IL-6 and TNF-α in each group (400 ×). (**B**) TUNEL results of apoptosis in each group (400 ×).. (**C**) Western blot results of HMGB1, IL-6 and TNF-α in each group. (**D**) SOD activity and MDA level in each group. (**E**) ROS production in each group. (**F**) Laser Doppler scanning of lower limb blood flow in each group. (**F**) Blood flow of lower limb in each group. All experiments were duplicated for three times. Values are mean ± standard deviation. *, *p* < 0.05. **, *p* < 0.01. ****, *p* < 0.0001.

Inflammation is one of the initial processes during the progressive phase of ischemia–reperfusion. Inflammatory factors such as HMGB1, IL-6 and TNF-α play an important role in the pathogenesis of ischemia–reperfusion in the arterial wall. Immunohistochemical staining of HMGB1, IL-6 and TNF-α showed that HMGB1, IL-6 and TNF-α were released from the nucleus and localized in the cytoplasm and interstitial space in the ischemia–reperfusion group, which fully demonstrated that ischemia–reperfusion disrupted the structural integrity of the cell membrane, cell swelling, increased membrane permeability, nuclear disintegration, and cell necrosis. Compared with IRI group, the expression levels of HMGB1, IL-6 and TNF-α in IRI + miR-206 agomir group were significantly decreased, while the expression levels in IRI + miR-206 antagomir group were significantly increased (Fig. 3A). We evaluated the apoptosis of femoral artery wall cells in each group by TUNEL technique, and observed the ultrastructural changes of apoptosis under a light microscope and photographed them. Apoptotic cells showed nuclear chromatin condensation, margination, nuclear lysis, apoptotic body formation, and cell membrane shrinkage and nuclear membrane lysis. The results showed that apoptosis was increased during ischemia–reperfusion, inhibited by overexpression of miR-206, and further increased by knockdown of miR-206 (Fig. 3B). Western blot results showed that the expression levels of HMGB1, IL-6 and TNF-α in femoral artery tissue of rats in the IRI group were significantly higher, and the increased expression of HMGB1, IL-6 and TNF-α was blocked after overexpression of miR-206, while the expression levels of HMGB1, IL-6 and TNF-α were further increased after knockdown of miR-206 (Fig. 3C). SOD and MDA are important indicators reflecting the balance of tissue redox status. When the production and clearance of SOD and MDA are disturbed and the redox balance is broken after tissue injury, the cells are dysfunctional, resulting in irreversible damage, including respiratory chain interruption, mitochondrial dysfunction, DNA damage, and then cell edema, necrosis, and disintegration. SOD activity was significantly decreased, while MDA level was significantly increased in IRI compared with sham group, and the decrease of SOD activity and the increase of MDA level were reversed after overexpression of miR-206, while SOD activity was further decreased and MDA level was increased after knockdown of miR-206 (Fig. 3D). Furthermore, ROS production was significantly increased in IRI compared with sham group, and the increase of ROS production were reversed after overexpression of miR-206, while ROS production was further increased after knockdown of miR-206 (Fig. 3E). These data confirmed that miR-206 improved the redox status of the femoral artery wall. Additionally, blood flow of lower limb was significantly reduced in IRI compared with sham group, and the reduction of blood flow of lower limb was reversed after overexpression of miR-206, while blood flow of lower limb was further decreased after knockdown of miR-206 (Fig. 3F). In conclusion, miR-206 inhibits ischemia–reperfusion injury in rat lower limb via oxidative stress.

## Discussion

Acute lower limb ischemia (ALI) is mainly caused by sudden interruption of distal limb arterial blood flow caused by lower limb arterial thrombosis arterial injury, and arterial emboli, and is one of the common arterial diseases in vascular surgery, with myocyte necrosis, sensory and motor loss occurring 3 h after ischemia and irreversible injury 6 h later^5,13,14^, which has a high disability rate and mortality and seriously threatens human health and safety^15–17^.

After lower limb ischemia, timely restoration of blood supply is the key to ensure the recovery of normal function of ischemic tissue, but ischemic tissue restores blood oxygen while causing further tissue damage, which is called ischemia–reperfusion injury (IRI)^18^. Affected by ischemia–reperfusion injury, limbs can develop from mild injury to systemic injury with systemic inflammatory response syndrome, involving multiple organs, which seriously threatens the health and life of patients^19–22^. Therefore, it is important to investigate the regulatory mechanism of inflammatory signaling to elucidate the molecular mechanism of acute lower limb ischemia–reperfusion injury. Min^22^ and other studies have found that waterfall inflammatory response triggered by limb ischemia–reperfusion injury leads to the release of a large number of inflammatory mediators into the blood, including: interleukin family (IL), tumor necrosis factor-a (TNF-a), intercellular adhesion molecule (ICAM) and vascular cell adhesion molecule (VACM); TNF-a, IL and can stimulate endothelial cells to secrete mobility group box 1 (HMGB1), while HMGB1 co-incubation with endothelial cells can induce ICAM, VCAM and E-selectin; exogenous HMGB1 can also promote the release of TNF-a, MCP-1, IL-8 and G-CSF. Our study showed that HMGB1, IL-6 and TNF-α expression was significantly upregulated in femoral artery tissue in a rat lower limb ischemia–reperfusion model.

The miR-206 family belongs to myogenic miRNAs and was originally cloned and identified from human and mouse muscle tissues, and gene chip detection suggests that miR-206 expression presents some spatiotemporal specificity during skeletal muscle cell differentiation and maturation, which suggests that miR-206 may have a role in contributing to muscle differentiation, which provides a new idea for the study of the mechanism of ischemia–reperfusion injury. MiR-206 has been well studied in cardiovascular disease, and Shan et al^23^. first found that miR-206 expression was significantly higher in infarcted myocardial tissue of mice. Westendorp et al^24^. found that miR-206 plays an important role in the evolution of dilated cardiomyopathy, and the main mechanism is that miR-206 is involved in regulating E2F6 to inhibit E2F/Rb signaling pathway, which in turn causes the down-regulation of CX43. miR-206 also plays an important role during skeletal muscle development, and Amirouche et al^25^. found that transfection of miR-206 into mouse myoblast cell lines in serum-containing medium differentiated them. Nakasa et al^25^. found that local injection of muscle-specific miR-206 promoted muscle regeneration in a rat model of skeletal muscle injury. Although miR-206 has been increasingly studied during skeletal muscle development, few studies have been reported in limb ischemia–reperfusion injury. Our study suggests that miR-206 attenuates lower limb IRI by regulating the action of inflammatory factors, and its molecular mechanism will be further explored in the future.

In this study, we investigated the effect of miR-206 on inflammatory factors and its possible role in the development of lower limb IRI, providing new research ideas for the regulatory mechanism of lower limb IRI, and providing a certain theoretical basis for the treatment of lower limb ischemia–reperfusion injury after surgery or endovascular intervention.

## Data Availability

The datasets used and/or analysed during the current study available from the corresponding author on reasonable request.
